# Tribological Behavior of Titanium Alloy Treated by Nitriding and Surface Texturing Composite Technology

**DOI:** 10.3390/ma12020301

**Published:** 2019-01-18

**Authors:** Jiajie Kang, Mingzheng Wang, Wen Yue, Zhiqiang Fu, Lina Zhu, Dingshun She, Chengbiao Wang

**Affiliations:** 1 School of Engineering and Technology, China University of Geosciences, Beijing 100083, China; 15522632403@163.com (M.W.); cugbyw@163.com (W.Y.); fuzq@cugb.edu.cn (Z.F.); zhulina@cugb.edu.cn (L.Z.); shedingshun@163.com (D.S.); cbwang@cugb.edu.cn (C.W.); 2 National International Joint Research Center of Deep Geodrilling Equipment, Beijing 100083, China; 3 Key Laboratory of Deep Geodrilling Technology, Ministry of Land and Resources, Beijing 100083, China; 4 National United Engineering Laboratory for Advanced Bearing Tribology, Henan University of Science and Technology, Luoyang 471023, China

**Keywords:** titanium alloy, plasma nitriding, laser surface texturing, tribological behavior

## Abstract

This study experimentally investigated the effect of surface textures on the tribological mechanism of nitrided titanium alloy (Ti–6Al–4V). The titanium alloy samples were nitrided at various temperatures ranging from 750 to 950 °C for 10 h in a plasma nitriding furnace. Then, surface textures were fabricated on the polished titanium alloy and plasma nitrided samples by laser process system. The surface roughness, microhardness, and constitution of samples treated by single nitriding and samples treated by composite technology were characterized. The tribological properties of the samples were investigated on a CSM ball-on-disc tribometer. The results show that plasma nitriding effectively enhances the wear resistance of the substrate. The wear rate decreases first and then increases with the increase of nitriding temperature, and the wear rate reaches the minimum at 900 °C. However, the increase in roughness caused by nitriding treatment leads to an increase in the friction coefficient. It is found that surface textures can obviously reduce the friction coefficient of the nitrided titanium alloy. In addition, it can also reduce the wear rate of titanium alloys after nitriding at 900 and 950 °C. It can be concluded that the nitriding and surface texturing combined treatment can obviously reduce the friction coefficient and wear rate at the nitriding temperatures of 900 and 950 °C. This is attributed to the combined effect of high hardness of nitride layers and the function of micro-trap for wear debris of surface textures.

## 1. Introduction

Titanium and titanium alloys possess good comprehensive properties, such as low density, high specific strength, high temperature resistance, corrosion resistance, magnetic resistance, impact resistance, and good biocompatibility, having extensive application prospects in the aviation field [1,2]. However, the low hardness and poor tribological performance limit the applications of titanium alloys, such as the application of titanium alloy fasteners in aerospace [3,4]. A lot of surface engineering technologies were developed to improve the wear resistance of titanium, such as thermal oxidation [5], ion implantation [6,7], thermal spraying [8,9,10], surface nanocrystallization [11,12], plasma nitriding [13,14], and surface texturing [15,16,17]. Among the above technologies, plasma nitriding and laser surface texturing are widely applied to improve the tribological performance of titanium [18,19].

Plasma nitriding (PN) technology, which has the advantages of controllable thickness and structure of the nitride layer, good process repeatability, and wide range of nitriding temperatures, is very suitable for the modification of titanium alloy [20]. The formed TiN*_x_* layer, which has an outer compound layer and an inner diffusion layer, has high hardness and excellent wear resistance [21,22]. Compared with the traditional gas nitriding, ion nitriding has many advantages, such as cleanliness, no pollution, fast penetration, energy saving, small distortion, adjustable layer composition, and wide temperature range [23,24,25]. However, ion nitriding needs a higher treatment temperature and longer time, which affects the structure and properties of the titanium alloy matrix to a certain extent. She et al. [26] studied the effects of nitriding temperature on microstructures and tribological properties of plasma-nitrided titanium under vacuum conditions. The results show that the nitriding temperature, as a critical process parameter, has a detrimental effect on both the hardness and ductility of nitriding layers. With the increase of nitriding temperature, the hardness increases gradually, but the ductility decreases. Thus, suitable nitriding temperature has an important influence on the friction properties of titanium alloy. Ali et al. [27] investigated the effects of nitriding time and nitriding environment on the friction properties of the Ti–6Al–4V alloy and revealed that the tribological behavior is also greatly influenced by nitriding time and nitriding environment. The results showed that the samples nitrided in gas mixture environment exhibited higher hardness compared to the samples nitrided in pure nitrogen gas environment. The samples nitrided for 4 h exhibited higher hardness compared to the samples nitrided for 18 h, which has the lowest tangential force coefficient, attributed to their high hardness. The wear volume and specific wear rate of the plasma nitrided samples were lower than those of the un-nitrided samples.

Laser surface texturing (LST), as a clean and efficient non-contact surface treatment technology, has been widely used in many fields [28]. The microscale geometric patterns can be introduced on the material by LST. Qiu et al. [29] reported that the shape, geometry, and density of surface textures have significant effects on the tribological performance of textured slider bearings. LST can be used to design numerous texture patterns, such as grid, chaotic, dimple, and groove types. The types of surface textures resulted in different performances of the materials. The most commonly used geometrical pattern is circular dimple, due to its easy fabrication and low costs [30]. Braun et al. [31] prepared micro-dimple textures on the steel by LST, and the results showed that the dimple textures can reduce more than 80% of friction forces. Yamakiri et al. [32] performed dimple patterns on the Si_3_N_4_ by using a Q-switch YAG laser. They found that the dimple textures have an important function of trapping wear debris and reducing abrasive damages on the sliding surface. By trapping wear debris, abrasion can be minimized, further reducing the wear of the bearing materials and improving the service life of the bearing couple [30]. 

The previous work shows that the nitrided titanium alloy has a higher hardness, which is of great benefit to improve the service life [33], but the brittleness of nitriding layers results in the abrasive wear. Borghi et al. [34] studied tribological effects of surface texturing on nitriding steel for high-performance engine applications, and the results show that in “dry contact” configuration, for a normal load of 1N, friction coefficient is reduced to about 10% from untextured to textured surface. However, the effect of surface texturing and nitriding composite treatment on the tribological properties of titanium has not been deeply investigated. In this study, the titanium alloy samples were nitrided at 750, 800, 850, 900, and 950 °C, respectively, for 10 h in a plasma nitriding furnace. Then, surface textures were fabricated on the nitrided samples by laser process system. For comparison, surface textures were also prepared on the polished titanium alloy. The present work aims to study the combined effect of surface textures and nitriding on the tribological performance of titanium alloy.

## 2. Experimental Details

### 2.1. Materials

The titanium alloy TC4 sheets (Φ 45 mm × 3 mm) of Ti–6Al–4V were used for the substrate. The atomic composition is 6.110% Al, 4.030% V, 0.120% Fe, 0.080% O, 0.001% H, and 0.02% C and balance Ti. The titanium alloy sheets were ground and polished to obtain a surface roughness of *S*_a_ < 100 nm. The microhardness is approximately 425 HV.

### 2.2. Plasma Nitriding Treatment

LDM 1-100 plasma nitriding furnace was used as the anode, and the polished titanium sheets were the cathode. The nitriding treatment was performed at different temperatures of 750, 800, 850, 900 and 950 °C for 10 h in the atmosphere of NH_3_, with a pressure about 500~550 Pa. Finally, the titanium sheets were cooled to room temperature in the NH_3_ atmosphere.

### 2.3. Laser Surface Texturing Treatment

Surface textures with circular dimple shape were prepared on the polished titanium sheets and nitrided samples by LM-S-YLP20F laser process system with an active medium of Ytterbium doped fiber. Both the diameter and interval of circular dimples of surface textures are 300 μm, and the area density is 19.63%. In previous studies, surface textures with different circular dimple diameters and intervals were prepared on titanium alloys, and it was found that when the area density of circular dimple texture is 19.63%, the wear resistance of titanium alloy is the best. Meanwhile, when the circular dimple diameter and spacing are 300 μm, the friction coefficient is the lowest. The surface textures with this texture parameter can significantly improve wear resistance and decrease friction coefficient. The laser process parameters are as follows: a wavelength of 1064 nm, a pulse duration of 100 ns, a frequency of 20 kHz, an average power of 10 W, and a traverse speed of 800 mm/s. After laser texturing treatment, the textured samples were polished again.

### 2.4. Characterizations

The morphologies of surface and wear scars were examined by scanning electron microscope (SEM, ZEISS Merlin Compact (Jena, Germany), equipped with Oxford EDX-450 model EDS (Jena, Germany)) and 3D white light interferometer surface profile meter (Nano-Map-D, AEP Technology, Santa Clara, CA, USA). The surface roughness, three-dimensional profile, wear volume, and depth of surface textures were characterized by 3D white light interferometer surface profile meter (Nano-Map-D). The metallurgical structure of cross-section of nitrided samples, which was polished and etched in Kroll’s reagent (2% HF and 4% HNO_3_, Beijing, China), was observed by metallurgical microscope (OLYMPUS BX51M, Tokyo, Japan). The phase structures of the nitrided samples were determined by Bruker D8 Advance X-ray diffractometer (Cu-Kα radiation source, λ = 1.5406 Å, 2θ ranges from 30° to 100°, increment 0.02°/step, 0.25 s per step; Billerica, MA, USA). The hardness values of nitrided and untreated samples were measured by an automatic microhardness tester (MICROMET-6030, Buehler, Dusseldorf, Germany) at a load of 50 gf. The hardness of each sample was measured five times and the average value was obtained. 

### 2.5. Friction and Wear Test

Friction and wear tests were conducted using a ball-on-disc friction tribometer (CSM, Peseux, Switzerland) in a unidirectional sliding mode. Si_3_N_4_ balls with a diameter of 4 mm were chosen as the friction pair. Before tests, the samples and the balls were cleaned with alcohol and acetone in ultrasonic cleaner for 20 min. During the tests, the ball was fixed, and the sample was rotated at a speed of 300 rpm (linear velocity ~0.09 m/s). The diameter of wear scar was set as 6 mm. The tests were carried out in an atmosphere environment at room temperature for 1200 s, and the load was 3 N.

## 3. Results and Discussion

### 3.1. Surface Morphologies

**Figure 1:**
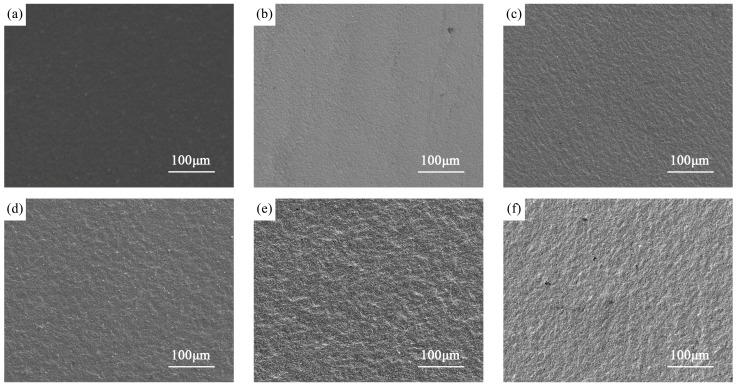
Surface morphology of titanium test samples after (**a**) polished (P), (**b**) plasma nitriding at 750 °C (750N), (**c**) plasma nitriding at 800 °C (800N), (**d**) plasma nitriding at 850 °C (850N), (**e**) plasma nitriding at 900 °C (900N), and (**f**) plasma nitriding at 950 °C (950N).

As shown in Figure 1, the surface roughness (*S*_a_, which equates to the arithmetical mean of the height deviations on the surface and reflects the deviation in height at each point from the arithmetic mean of the surface [35,36]) of the nitrided titanium alloy increases compared with that of the polished titanium alloy. In addition, the surface roughness of nitrided titanium alloy increases with the increase of the nitriding temperature. This is due to the formation of nitrides in the nitriding process. When the nitrides formed on the surface, some small bumps with different sizes were generated. With the increase of nitriding temperature, the nitrides formed on the surface gradually increase, so that the surface roughness of the nitrided sample tends to increase gradually with the increase of nitriding temperature [22,26].

**Figure 2:**
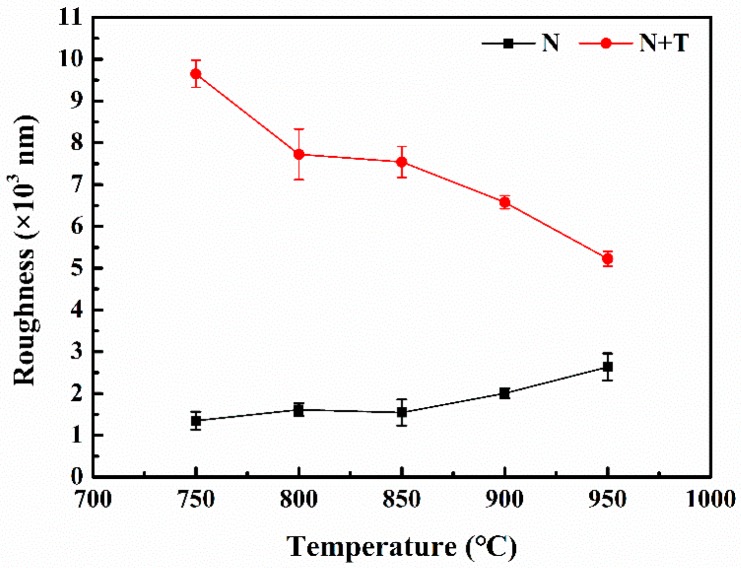
Surface roughness and three-dimensional morphology of titanium alloy samples after nitriding and nitriding/texturing combined treatment at different temperatures.

**Figure 3:**
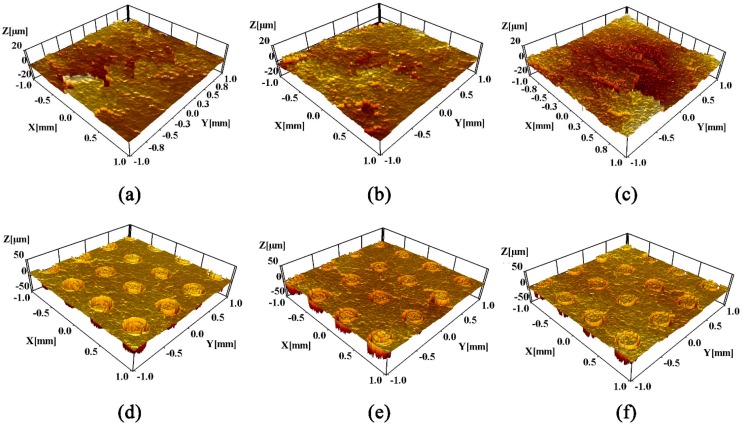
Three-dimensional morphology of titanium alloy samples after nitriding and nitriding/texturing combined treatment at (**a**,**d**) 750 °C, (**b**,**e**) 850 °C, (**c**,**f**) 950 °C.

The surface roughness and three-dimensional morphologies of the titanium alloy samples after nitriding, and nitriding/texturing combined treatments at different temperatures, are shown in Figure 2 and Figure 3. The surface roughness of the nitrided sample increases with the increase of the nitriding temperature for the reason mentioned above, but on the contrary, the surface roughness of the titanium alloy after nitriding/texturing combined treatments decreases with increasing nitriding temperature. This is because that texture-induced changes in roughness are much greater than those caused by nitriding, and dominate the changes in roughness. After nitriding, a layer of nitride is formed on the titanium alloy surface. The thermal conductivity of this layer is higher than that of the matrix *k*_Ti_ < *k*_TiN_ (the thermal conductivity of TiN is 29.31 W m^−1^ K^−1^ at room temperature, and the thermal conductivity of titanium is 15.24 W m^−1^ K^−1^ [37,38]). As a result, heat conduction away from the surface proceeds slower for titanium than TiN in the laser etching process. Thus, a larger fraction of the laser energy is retained close to the incident surface, leading to a rapid rise in surface temperature, while the bulk material remains relatively cool. Therefore, titanium alloy is etched more than TiN [39], which can also be seen in Figure 3. Therefore, the surface roughness of the samples after nitriding/texturing combined treatment decreases as the nitriding temperature increases.

### 3.2. Structural Characteristics of the Nitrided Samples 

**Figure 4:**
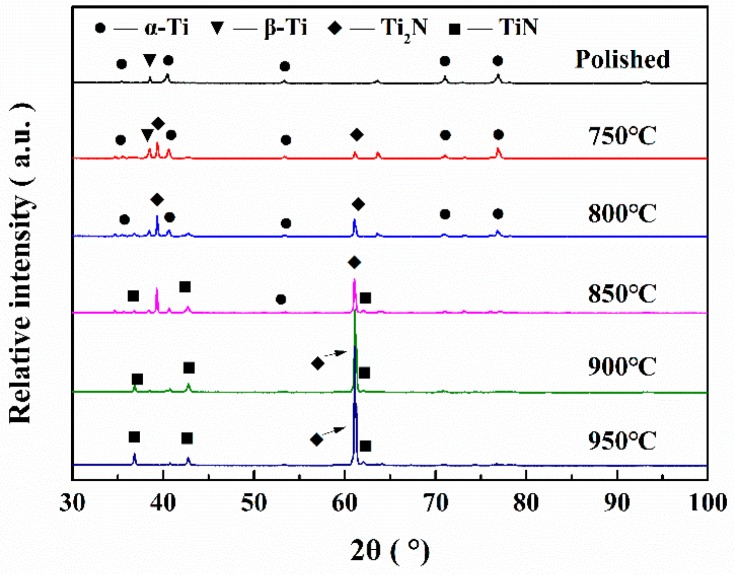
XRD patterns of titanium samples at different nitriding temperatures.

Figure 4 shows the XRD patterns of titanium alloys at different nitriding temperatures. The titanium alloy mainly consists of α-Ti and β-Ti phases before nitriding. After nitriding, TiN, Ti_2_N, and other hard titanium nitride phases formed on the titanium alloy. The Ti_2_N phase was generated when nitriding at 750 and 800 °C, while the Ti_2_N phase and TiN phase were formed on the titanium alloy when nitriding at 850, 900 and 950 °C.

**Figure 5:**
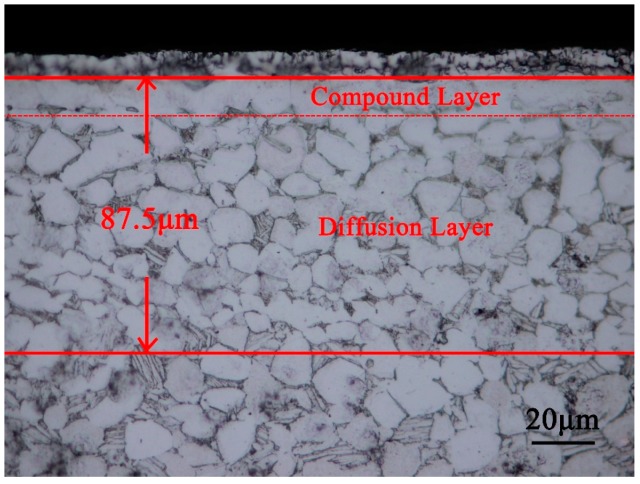
Cross-section microstructure of titanium alloy samples after nitriding treatment.

Figure 5 shows the cross-section structure of nitrided samples. The nitride layer consists of a white compound layer of titanium nitride and a diffusion layer composed of white equiaxed crystals [20]. The thickness of the nitride layer is about 87.5 μm. As the nitriding temperature increases, the nitrogen diffusion in the matrix and the reaction rate increases, which makes the thickness of the diffusion layer and compound layer increase [26].

**Figure 6:**
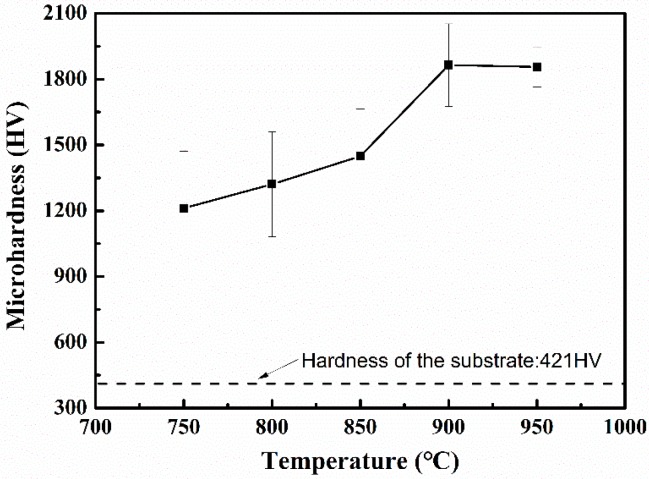
Microhardness of titanium alloy samples at different nitriding temperatures.

Figure 6 shows the microhardness of titanium alloy samples at different nitriding temperatures. The microhardness of the titanium alloy surface after nitriding is significantly increased relative to that of the titanium alloy substrate (421 HV). In addition, as the nitriding temperature increases, the microhardness gradually increases. The microhardness reaches the maximum of 1856 HV at the nitriding temperature of 950 °C. This is associated with the increase of the hard phase of the titanium nitride in the titanium alloy as the nitriding temperature increases (see Figure 4). In addition, a bright nitride layer can be seen on the surface of the titanium alloy after nitriding treatment in Figure 5. Figure 6 shows that more nitride layers begin to appear when nitriding temperature is 900 and 950 °C. The nitride layer on the surface has a significant effect on the measurement of microhardness, resulting in a similar microhardness of the surface of the titanium alloy after nitriding at 900 and 950 °C.

It is worth mentioning that the heat affected zone created by the laser surface texture can also increase the hardness of the substrate. However, the increase in hardness (maximum 38% increase) due to the laser surface texture is much smaller than the increase in hardness (maximum 340% increase) due to nitriding, so the heat affected zone produced by the laser surface texture can be temporarily ignored [40]. 

### 3.3. Frictional Properties

**Figure 7:**
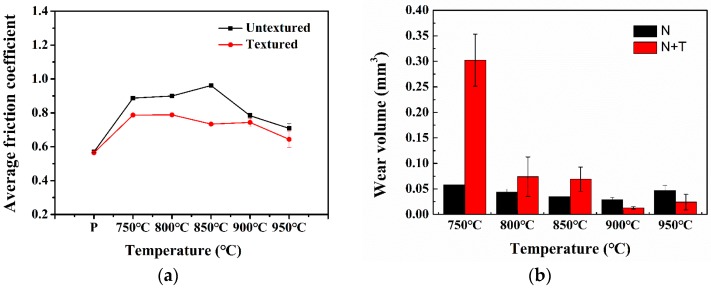
(**a**) Friction coefficient and (**b**) wear volume after nitriding and nitriding/texturing duplex treatment at different nitriding temperatures.

Figure 7a shows the relationship between the friction coefficient and nitriding temperatures for the samples after nitriding and nitriding/texturing combined treatment. The friction coefficient of the polished titanium alloy is almost the same as that of the textured titanium alloy, and is lower than the friction coefficient of the titanium alloy after nitriding treatment and nitriding/texturing combined treatment. This is mainly due to the fact that the hardness of surface textures on the titanium alloy (about 421 HV) is much lower than that of Si_3_N_4_ (about 1600 HV), resulting in the failure of the surface textures. Thus, the grinding ball directly contacts with the matrix. The surface of the polished titanium alloy is smooth, and the main wear mechanism is abrasive wear, which leads to the relatively low friction coefficient. For the nitrided titanium alloy, the friction coefficient increases first and then decreases with the increase of nitriding temperature. This is mainly because the surface roughness and hardness increases gradually as the nitriding temperature increases, which increases the wear of the grinding ball. Therefore, the friction coefficient increases gradually with the increase of nitriding temperature due to more severe friction. On the other hand, with the increase of nitriding temperature, the microhardness of titanium alloy is effectively improved, but the brittleness of nitride layer also increases [26]. In the process of friction, the hard abrasive debris will more likely spall, resulting in the transformation of the wear mechanism from adhesion to abrasive wear, which reduces the friction coefficient. As a result of these two reasons, the friction coefficient increases first and then decreases with the increase of nitriding temperature. However, the friction coefficient of nitriding/texturing treated titanium alloy gradually decreases with the increase of nitriding temperature, which is mainly because the surface textures can play the role of storing debris [41,42]. Therefore, the hard debris is not involved in the friction process. Moreover, the surface textures after the combined treatments show relatively high hardness, and they will not fail easily during the friction process. Therefore, the surface roughness plays a leading role in the increase of friction coefficient. The surface roughness of the titanium alloy after combined treatment decreases with the increase of nitriding temperature, which results in the reduction of friction coefficient. Overall, the friction coefficient of titanium alloy after combined treatment is lower than that of nitrided titanium alloy, which indicates that surface textures can significantly reduce the friction coefficient of nitrided titanium alloy. 

Figure 7b shows the wear volumes of nitrided titanium alloy (N) at different nitriding temperatures and samples (N + T) after nitriding/texturing combined treatments. In Figure 7b, with the increase of nitriding temperature, the wear volume of both N and N + T first decreases and then increases. The wear volume of N + T at 900 °C is the least. When nitriding temperature is between 750 and 850 °C, the wear resistance of N is better than that of N + T. However, at the high temperatures of 900 and 950 °C, the wear resistance of N + T is superior than that of N. This is because both the hardness and brittleness of the nitrided layer on the titanium alloy increases with the increase of nitriding temperature. When the nitriding temperature is lower, the hardness is improved but the wear debris is relatively less. Thus, the wear volume decreases with the increase of the nitriding temperature. However, when the nitriding temperature is 950 °C, the abrasive wear caused by the spalling hard abrasive debris is more serious, which increases the wear volume. In addition, N + T decreases the surface contact area, which results in the increase of contact stress. When nitriding temperature is lower (750–850 °C), the nitride layer is thinner and the hardness is lower. In addition, the texture increases the contact stress. During the friction process, the composite treated nitriding layer will be more prone to break off than that of N alone, so the wear volume of N + T will be larger than that of N. When the nitriding temperature is higher, the nitride layer is thick enough, and the hardness is large enough, so that it is more difficult to break off during the friction process. Moreover, surface textures can also play the role of storing the hard debris during the friction process, reducing abrasive wear and making the wear volume smaller than that of the nitrided sample.

### 3.4. The Wear Mechanism

**Figure 8:**
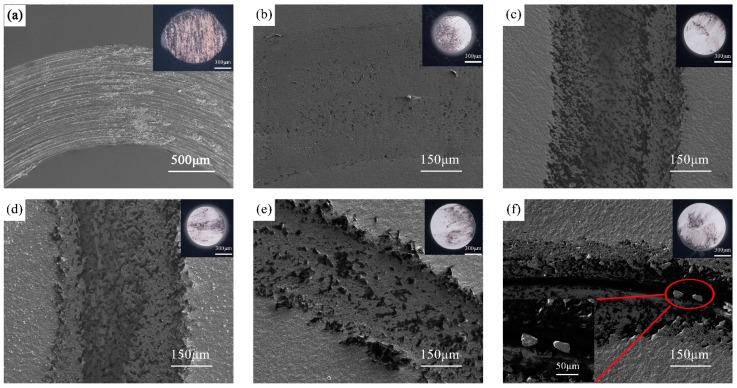
Wear scar morphologies of grinding ball and titanium alloy after (**a**) polishing and nitriding at (**b**) 750 °C, (**c**) 800 °C, (**d**) 850 °C, (**e**) 900 °C, and (**f**) 950 °C.

Figure 8 shows the wear scar morphologies of grinding ball and titanium alloy after polishing and nitriding at different temperatures. As can be seen from Figure 8a, there are many furrows and slices of debris on the wear scar surface of the polished titanium alloy and grinding ball, thus the wear mechanism is mainly abrasive wear and adhesive wear. This is because the hardness of the titanium alloy is much lower than that of the grinding ball. In addition, the oxide film was generated continuously in the friction interface of the atmosphere and finally fell off. The detached oxide film caused severe abrasive wear during the friction process. From Figure 8b–f, as the nitriding temperature increases, the wear debris on the wear scar surface increases. This is mainly because the surface roughness and hardness gradually increased with the increase of nitriding temperature, and the wear of the grinding ball became more serious (increase in wear spot diameter), which caused the wear debris to increase with the increase of nitriding temperature. In addition, it is noteworthy that in Figure 8f, severe furrows occurred in the wear scars due to wear debris. The wear mechanism has changed from the adhesive wear at the lower nitriding temperature due to serious abrasive wear. As mentioned before, as the nitriding temperature increases, the brittleness gradually increases, resulting in spalling and participating in the friction of wear debris in the titanium alloy sample nitrided at 950 °C. When the nitriding temperature is below 950 °C, the hardness of the nitrided titanium alloy increases and the brittleness is not high enough to generate hard wear debris, so that the main friction mechanism is mainly the adhesive wear.

**Figure 9:**
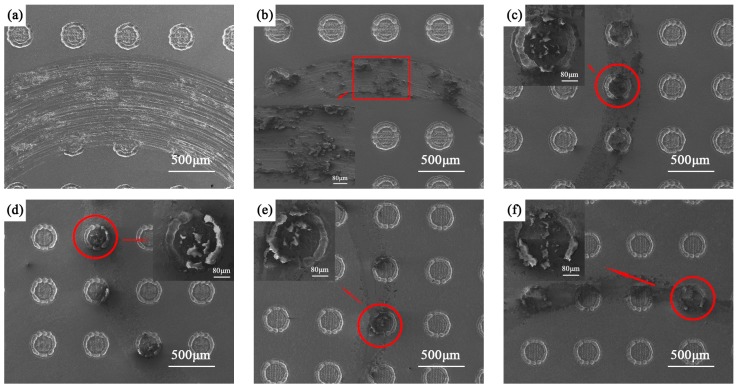
Wear morphologies of titanium alloys after duplex treatment at different temperatures. (**a**) Polished + T, (**b**) 750N + T, (**c**) 800N + T, (**d**) 850N + T, (**e**) 900N + T, and (**f**) 950N + T.

The wear morphologies of titanium alloys after combined treatment at different temperatures are shown in Figure 9. In Figure 9a, there are obvious furrows and wear debris on the wear scars, which indicates that the wear mechanism of the textured titanium alloy is mainly abrasive wear and adhesive wear. This is mainly because the hardness of titanium alloy is lower, and the contact stress increases after texturing treatment. During the friction process, the surface textures will become invalid in a relatively short time, and it will not play an obvious role in reducing wear volume and friction coefficient. Figure 9b shows that after the texturing treatment, the spalling of nitride layer occurs in the wear scar. This is mainly because that the thickness and hardness of the nitride layer on the surface of the titanium alloy after nitriding at 750 °C are relatively small. The surface textures also make the contact stress larger, so that the nitride layer spalls during the friction process, which also leads to a higher wear amount of the combined treated sample at 750 °C than that at other temperatures higher than 750 °C. From Figure 9c–f, the wear scars of the combined treated samples at 800, 850, 900, and 950 °C are much smoother than that of the nitrided sample, and partial wear debris is captured by surface textures. Therefore, the debris no longer participates in the friction and the adhesive of titanium after nitriding at 800–900 °C, nor do furrows occur at 950 °C. This indicates that the surface textures can play the role of storing wear debris and reducing wear.

## 4. Conclusions

The plasma nitriding and nitriding/texturing treatments were performed on the titanium alloy (Ti–6Al–4V) at various temperatures ranging from 750 to 950 °C. The effect of nitriding/texturing combined treatment on tribological properties of titanium was experimentally investigated. On the basis of our findings, the conclusions are as follows:

- The microhardness of the titanium alloy was significantly improved after nitriding. With the increase of nitriding temperature, the nitride content of the nitriding layer on the surface of the titanium alloy increases and the surface hardness also increases. However, at the same time, the brittleness and roughness of the nitride layer also increase.
- Due to the increase of hardness, brittleness, and roughness of nitriding layer, the friction coefficient of nitrided titanium alloy increases first and then decreases with the increase of nitriding temperature. The wear volume tends to decrease first and then increase with the increase of nitriding temperature.
- After the surface texture treatment, the friction coefficient of nitrided titanium alloy samples decreases in varying degrees. When the nitriding temperature is lower (750~850 °C), the wear volume after the combined treatment is larger than that after the nitriding treatment. When the nitriding temperature is higher (900~950 °C), the wear volume of samples after the combined treatment is less than that of the nitrided sample. This indicates that when the nitriding temperature is higher, the surface texture can play a role in improving the wear resistance of the nitrided titanium alloy.
- The wear mechanism of the titanium alloy sample after nitriding is mainly the adhesive wear and the abrasive wear. The adhesive wear dominates when the nitriding temperature is low, while abrasive wear dominates when the nitriding temperature is high. The wear mechanism of the titanium alloy sample after nitriding/texture combined treatment is mainly adhesive wear. The surface textures prepared on the surface of the nitrided titanium alloy with high hardness show the ability of storing wear debris. Therefore, it can effectively increase the wear resistance of the titanium alloy.
